# A SYSTEMATIC REVIEW OF EFFECTIVENESS OF TECHNOLOGY INTERVENTIONS FOR PEOPLE WITH DEMENTIA AND INFORMAL CAREGIVERS

**DOI:** 10.1093/geroni/igad104.1598

**Published:** 2023-12-21

**Authors:** Ling Xu, Lingya Zhao

**Affiliations:** The University of Texas at Arlington, Arlington, Texas, United States; Capital University of Economics and Business, Beijing, Beijing, China (People’s Republic)

## Abstract

**Objectives:**

Most people with dementia live at home and get care from informal caregivers. To support people with dementia and informal caregivers, a series of technology interventions have been introduced and implemented. The aim of this study was to evaluate effectiveness of recent technology interventions. Method: A systematic literature search was carried out in 14 online databases and Google Scholar, covering articles published during January 2005 and January 2020. Over three hundred articles were identified, of which 23 met the inclusion criteria and were included in the review. Meta-analyses for characteristics of included studies were conducted.

**Results:**

Most studies demonstrated that technology interventions had various benefits for both people with dementia and their informal caregivers living in the community. For informal caregivers, these interventions tend to improve their psychological conditions and provide useful help when caring for people with dementia. For people with dementia, these interventions have potential to improve their performance and mental conditions, and keep them safe at home, which could delay institutionalization and save financial costs.

**Conclusion:**

Technologies can be used independently by people with dementia, to help them in positive psychological condition, and to maintain their basic social interaction should be developed. Technology interventions should be developed and selected according to the progression of dementia. More assessment studies using randomized controlled trails should be conducted, so that we can evaluate extant technology interventions more rigorously.

